# The LIFE-Adult-Study: objectives and design of a population-based cohort study with 10,000 deeply phenotyped adults in Germany

**DOI:** 10.1186/s12889-015-1983-z

**Published:** 2015-07-22

**Authors:** Markus Loeffler, Christoph Engel, Peter Ahnert, Dorothee Alfermann, Katrin Arelin, Ronny Baber, Frank Beutner, Hans Binder, Elmar Brähler, Ralph Burkhardt, Uta Ceglarek, Cornelia Enzenbach, Michael Fuchs, Heide Glaesmer, Friederike Girlich, Andreas Hagendorff, Madlen Häntzsch, Ulrich Hegerl, Sylvia Henger, Tilman Hensch, Andreas Hinz, Volker Holzendorf, Daniela Husser, Anette Kersting, Alexander Kiel, Toralf Kirsten, Jürgen Kratzsch, Knut Krohn, Tobias Luck, Susanne Melzer, Jeffrey Netto, Matthias Nüchter, Matthias Raschpichler, Franziska G. Rauscher, Steffi G. Riedel-Heller, Christian Sander, Markus Scholz, Peter Schönknecht, Matthias L. Schroeter, Jan-Christoph Simon, Ronald Speer, Julia Stäker, Robert Stein, Yve Stöbel-Richter, Michael Stumvoll, Attila Tarnok, Andrej Teren, Daniel Teupser, Francisca S. Then, Anke Tönjes, Regina Treudler, Arno Villringer, Alexander Weissgerber, Peter Wiedemann, Silke Zachariae, Kerstin Wirkner, Joachim Thiery

**Affiliations:** LIFE - Leipzig Research Centre for Civilization Diseases, University of Leipzig, Leipzig, Germany; Institute for Medical Informatics, Statistics, and Epidemiology (IMISE), University of Leipzig, Haertelstrasse 16-18, 04107 Leipzig, Germany; Department of Sport and Exercise Psychology, University of Leipzig, Leipzig, Germany; Max Planck Institute for Human Cognitive and Brain Sciences, Leipzig, Germany; Clinic of Cognitive Neurology, University of Leipzig, Leipzig, Germany; Institute of Laboratory Medicine, Clinical Chemistry and Molecular Diagnostics, University of Leipzig, Leipzig, Germany; Department of Internal Medicine/Cardiology, Leipzig Heart Centre, Leipzig, Germany; Interdisciplinary Centre for Bioinformatics, University of Leipzig, Leipzig, Germany; Department of Psychosomatic Medicine and Psychotherapy, Universal Medical Centre Mainz, Mainz, Germany; Department of Otorhinolaryngology, Section of Phoniatrics and Audiology, University of Leipzig, Leipzig, Germany; Department of Medical Psychology and Medical Sociology, University of Leipzig, Leipzig, Germany; Department of Cardiology-Angiology, University of Leipzig, Leipzig, Germany; Department of Psychiatry and Psychotherapy, University of Leipzig, Leipzig, Germany; Clinical Trial Centre Leipzig - Coordinating Centre for Clinical Trials, University of Leipzig, Leipzig, Germany; Department of Electrophysiology, Leipzig Heart Centre, Leipzig, Germany; Clinic of Psychosomatic Medicine and Psychotherapy, University of Leipzig, Leipzig, Germany; Interdisciplinary Centre for Clinical Research (IZKF), University of Leipzig, Leipzig, Germany; Institute of Social Medicine, Occupational Health and Public Health (ISAP), University of Leipzig, Leipzig, Germany; Department of Pediatric Cardiology, Leipzig Heart Centre, Leipzig, Germany; Department of Radiology, University of Leipzig, Leipzig, Germany; Department of Ophthalmology, University of Leipzig, Leipzig, Germany; Department of Dermatology, Venereology and Allergology, University of Leipzig, Leipzig, Germany; Medical Department, Division of Endocrinology and Nephrology, University of Leipzig, Leipzig, Germany

**Keywords:** Cohort study, Epidemiology, Population based, Diseases of civilization

## Abstract

**Background:**

The LIFE-Adult-Study is a population-based cohort study, which has recently completed the baseline examination of 10,000 randomly selected participants from Leipzig, a major city with 550,000 inhabitants in the east of Germany. It is the first study of this kind and size in an urban population in the eastern part of Germany. The study is conducted by the Leipzig Research Centre for Civilization Diseases (LIFE). Our objective is to investigate prevalences, early onset markers, genetic predispositions, and the role of lifestyle factors of major civilization diseases, with primary focus on metabolic and vascular diseases, heart function, cognitive impairment, brain function, depression, sleep disorders and vigilance dysregulation, retinal and optic nerve degeneration, and allergies.

**Methods/design:**

The study covers a main age range from 40-79 years with particular deep phenotyping in elderly participants above the age of 60. The baseline examination was conducted from August 2011 to November 2014. All participants underwent an extensive core assessment programme (5-6 h) including structured interviews, questionnaires, physical examinations, and biospecimen collection. Participants over 60 underwent two additional assessment programmes (3-4 h each) on two separate visits including deeper cognitive testing, brain magnetic resonance imaging, diagnostic interviews for depression, and electroencephalography.

**Discussion:**

The participation rate was 33 %. The assessment programme was accepted well and completely passed by almost all participants. Biomarker analyses have already been performed in all participants. Genotype, transcriptome and metabolome analyses have been conducted in subgroups. The first follow-up examination will commence in 2016.

## Background

Since the German reunification in 1990, East Germany has experienced profound social and lifestyle changes. Large efforts have been undertaken to equalize the previous disparities of living conditions between East and West Germany. Remarkably, the disparities of life expectancy of about 2-3 years observed in the 1990s have vanished within 20 years [1]. However, differences in health related aspects still exist. Standardized mortality rates due to cardiovascular diseases are still high in the east German states of Saxony-Anhalt, Mecklenburg-Western Pomerania, and Saxony. The prevalence of psychiatric disorders is increasing both in the western and eastern part of Germany, however, at a faster rate in the east. The eastern states of Germany also face a progressive demographic change. Therefore, disease prevention programmes and predictions of disease developments are urgently needed by various stakeholders in the German health care system.

Population-based cohort studies are a vital means to investigate the interactions of predisposing genetic, environmental, social and lifestyle factors with health maintenance and disease onset. Several population-based studies have been conducted in other areas of Germany in the past decades focusing on a limited set of phenotypes and specific diseases [2–6]. In 2009, the Leipzig Research Centre for Civilization Diseases (LIFE) was initiated within the highly competitive intitiative for excellence in research of the Free State of Saxony. LIFE is part of the Medical Faculty of the University of Leipzig, enabling participation of clinical and epidemiological researchers experienced in the field of metabolic, neurological, cardiovascular, allergological and ophthalmological disorders, and of methodological researchers experienced in molecular and genetic profiling, biostatistics and bioinformatics. In addition, researchers of the Leipzig Heart Centre and the Max Planck Institute for Human Cognitive and Brain Sciences Leipzig, two major medical research institutions in the city, are involved in the LIFE-Adult-Study.

Leipzig is a city with approximately 550,000 inhabitants of mostly central European descent. The LIFE-Adult-Study pursues the following major goals: i) to assess the prevalence and incidence of common diseases and subclinical disease phenotypes, ii) to investigate the complex interactions between molecular-genetic and lifestyles factors regarding co-occurrence and development of subclinical phenotypes and diseases, and iii) to investigate in a longitudinal extension of the study the role of biomarkers to predict risks for disease progression. In particular, we focus on the following mostly age-related clinical phenotypes: metabolic disorders (type 2 diabetes, hyperlipoproteinemia, obesity), cardiovascular disorders and heart failure, cognition and brain function, depression, sleep disorders and vigilance regulation, early signs of retinal and optic nerve degeneration, and allergies (Table 1). We introduced several innovative assessment techniques in order to investigate novel phenotypes, e.g. optical coherence tomography (OCT) of the retina, 3D body surface scans, EEG-based recording of vigilance regulation, sleep duration, voice function. This is accompanied by analyses of more than 80 clinical chemical biomarkers, and comprehensive molecular-genetic profiling comprising genotyping, transcriptome, metabolome, and proteome analyses.

**Table 1:** Major diseases and phenotypes investigated in the LIFE-Adult-Study

●	Cardiovascular diseases (myocardial infarction, heart failure, atherosclerosis, cardiac arrhythmias)
●	Metabolic diseases (obesity, type 2 diabetes)
●	Cognition and brain functioning
●	Depression (including minor depression)
●	Sleep disorders and vigilance regulation
●	Degenerative diseases of the retina
●	Allergies and immune competence

The study was designed in 2008. Funding was available in August 2009. Positive ethics votes were obtained in 2010. Feasibility studies for several assessments started late in 2010. A pilot study to optimize the full programme was conducted early in 2011, when the biorepository and the IT-infrastructure was established. A comprehensive set of standard operating procedures (SOP) was implemented. Recruitment started in August 2011. In November 2014 the recruitment of the cohort was successfully completed.

This paper describes the objectives and the design of the LIFE-Adult-Study. We also present first results regarding the recruitment, response rates, and conduct of the study.

## Methods/Design

### Study population and recruitment

**Fig. 1:**
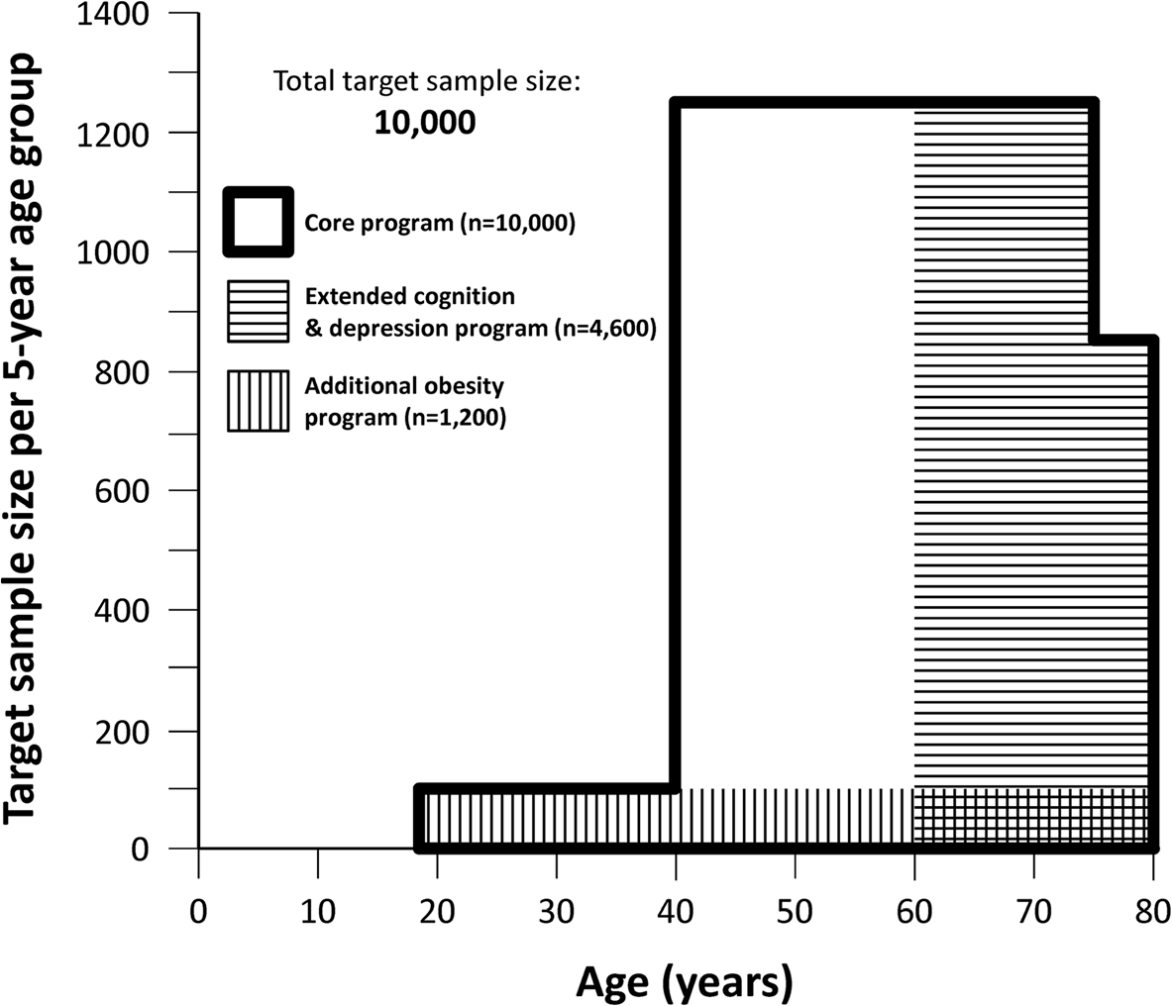
Target sample sizes of the LIFE-Adult-Study

The study design comprised an age and gender stratified random sample of residents of the city of Leipzig, in the age group of 40 to 79 years. A subset of 400 participants aged 18 to 39 years was also recruited. In total, 10,000 participants were planned as the target population (Fig. 1). Address lists of randomly sampled citizens were provided by the resident’s registration office of the city of Leipzig. Citizens were sent an invitation letter containing an information leaflet about the study, a response form and a postage-paid return envelope. Persons who did not respond within four weeks received a reminder letter. Non-responders were searched in public telephone directories and contacted by phone. Persons who were interested to participate were scheduled for an appointment in the LIFE study centre. As a prerequisite to enrolment, written informed consent was obtained from all participants. The study was approved by the responsible institutional ethics board of the Medical Faculty of the University of Leipzig. The data privacy and safety concept of the study was approved by the responsible data protection officer. Possible incidents during study visits and during travel to the study site were covered by an insurance policy. Participants received a lump sum of 20 EUR per visit to cover their travel expenses. No other financial incentives were paid out. We carried out several public relation activities to stimulate participation rates. The LIFE study centre was located on the medical campus in the centre of the city, which was easy to reach.

In March 2013, we decided to modify the inclusion criteria of the programme with a reduction of the upper age limit to 74 years. We observed that participants aged ≥75 years had difficulties in completing the assessment programme within the set time limit despite high motivation. Furthermore, it became apparent that participation of women in this age group was markedly reduced (about 1/3 lower than men). The most frequently given reason was that women would not leave their diseased and care-needing partners alone at home on three study days. Therefore, as we stopped recruitment of participants ≥75 years, we extended the lower age limit for deep cognition and depression phenotyping to 60 years. This change was put in place in March 2013 after approval by the institutional ethics board.

In a subset of participants we investigated whether body fat distribution is associated with functional traits of the brain (magnetic resonance imaging, MRI) and traits of eating behaviour. To unravel this question a subcohort of 1200 participants aged 18-79 underwent abdominal MRI-scans in addition to brain MRI-scans.

### Disclosure of results and pathological findings to participants

Participants were offered to be informed about selected examination results. If they agreed, they received a letter within two weeks of the study visit comprising their laboratory and anthropometric data, blood pressure, intima media thickness, ankle-brachial index, and the results of the skin prick test. Participants were informed about pathological findings according to a pre-defined protocol using four categories of severity (very high: acute life-threatening condition requiring immediate intervention, high: not acute life-threatening condition needing immediate medical clarification or treatment, medium: need for near-term medical clarification, low: recommendation of medical supervision, e.g. by a general practitioner).

### Data management

We developed and set up a comprehensive dedicated IT infrastructure to support processes such as recruiting, appointment scheduling, assessment coordination, data entry, data integration, pre-processing, quality control, data curation and analysis. The infrastructure consists of several commercial and self-developed software systems running in a shared network. An ORACLE® database (Oracle Corporation, Redwood Shores, CA, USA) is used for storing administrative and most assessment data. A tailor-made participant management system was used to organise participant data (e.g. subject identity, pseudonymisation, and appointments) separately from the scientific data. LimeSurvey® (www.limesurvey.org) and TeleForm® (Electric Paper Informationssysteme GmbH, Lüneburg, Germany) were used to collect data from interviews, questionnaires and examinations via PC-based or paper-based data capture, respectively. Dedicated IT processes were put in place to collect image and biosignal data from measurement devices wherever possible. All biospecimen related processes (e.g. collection, labelling, processing, aliquoting, distribution, storage) were administered using a self-developed laboratory information system (LIMS) - CryoLab. Data and analysis results were integrated into a comprehensive research database. We used the ‘LIFE Investigation Ontology’ (LIO) to semantically describe, classify and retrieve data in the research database [7].

### Biobanking

A novel semi-automated cryobiobank with continuous cooling chains warrants sustainability for later bioanalytics. In the LIFE-Adult-Study, biospecimen of stabilized (Tempus, life technologies) and unstabilized whole blood (EDTA-blood), dried blood spot cards, serum, plasma (EDTA, citrate, lithium heparine), urine, peripheral blood mononuclear cells (PBMC), DNA and RNA samples were collected and stored according to SOPs. 50 different aliquots were cloned within 2 h of blood sampling and stored at -80 °C (DNA, RNA, EDTA-blood, urine, serum) or at temperatures below -150 °C (serum, plasma, PBMCs) in the gas phase of liquid nitrogen. Prior to storage, samples were processed and cloned in barcode labelled cryotubes and straws. To minimize freeze-thaw cycles, samples were sorted in a cryogenic work bench (temperatures below -100 °C) and automatically stored in tanks with a coolable top frame in the gas phase of liquid nitrogen (Askion, Germany).

### Quality management

Prior to the study, several assessments were tested in feasibility studies in order to evaluate their practicability, time requirements, inter- and intra-observer reliability, and validity [8–10]. The entire assessment programme was tested in a pilot study with approximately 400 probands. For all assessments, SOPs were established by teams of at least one epidemiologist and one clinical expert. Adherence to SOPs was reviewed by appointed internal supervisors in regular intervals. The conduct of the assessment programmes was audited by external experts. All staff members underwent extensive training before conducting assessments. After data collection, data were checked regularly for completeness, plausibility and consistency using pre-defined algorithms. Furthermore, data were monitored statistically at the population level in order to detect deviations from SOP adherence or malfunctions of medical devices.

### Assessment programme

The assessment programme comprised physical and medical examinations (Table 2), computer-assisted personal interviews, computer- or paper-based self-administered questionnaires, psychometric tests (Table 3), and clinical chemistry from blood and urine samples (Table 4). All study participants underwent a core assessment programme with an average duration of 5-6 h within one day (‘core programme’). Participants aged ≥65 years (≥60 years and above since March 2013) were invited to take part in two additional assessment programmes, which were scheduled shortly after the core programme visit on two separate days with an average duration of 3-4 h each. One of these additional programmes focussed on cognitive function and a MRI scan of the brain (‘cognition programme’). The second additional programme was dedicated to detailed assessment of depressive symptoms and vigilance including multi-paradigm EEG (‘depression programme’).

**Table 2:** Physical and medical examinations

Examination	Sample size
Bio-specimen asservation	
Serum, plasma, lymphocytes for DNA, RNA, urine	>9900
Anthropometry	
Classical: Body weight, body height, circumference measures	>9900
3D laser based optical body surface scan	>9800
MRI based abdominal fat tissue volumetry	>1000
Cardiovascular system	
Blood pressure	>9900
Electrocardiography (10 s, 12 leads)	>9700
Echocardiography	>8300
Carotid ultrasound	>9600
Ankle-brachial index (ABI), pulse wave velocity (PWV)	>9200
Diabetes	
Oral glucose tolerance test (OGTT)	>2900
Physical activity and fitness	
Hand grip strength	>9700
7 day actimetry	>2800
Eye	
Optical coherence tomography and fundus photography	>9300
Brain	
MRI	>2400
Electroencephalography (≥60 years only)	>3000
Miscellaneous	
Voice profile	>2700
Olfactory test (Sniffin’ sticks 12)	>7300
Skin prick test (six inhalative allergens)	>8000

**Table 3:** Computer- assisted personal interviews (I), self-administered questionnaires (Q) and cognitive tests (T)

Assessment	Type	Target population (age)
Socio-demographics and medical history		
Socio-demographics and socio-economic status	I	18-79
Medical history	I	18-79
Family medical history	I	18-79
Medication (last 7 days)	I	18-79
Allergies	I	18-79
Male and female gender questions	Q	18-79
Immune competence	Q	18-79
Oral health	Q	18-79
Life style		
Smoking	I/Q	18-79
Food frequency and alcohol consumption	Q	18-79
International Physical Activity Questionnaire (IPAQ)	Q	18-79
Three-Factor-Eating Questionnaire (TFEQ)	Q	18-79
Yale Food Scale	Q	18-79
Psychosocial aspects		
Satisfaction with Life Scale (SWLS)	Q	18-79
Generalized Anxiety Disorder (GAD-7)	Q	18-79
Patient Health Questionnaire (PHQ-15)	Q	18-79
SF-8 Health Survey	Q	18-79
Life Orientation Test Revised (LOT-R)	Q	18-79
ENRICHD Social Support Instrument (ESSI)	Q	18-79
Personality Adjective List (16 AM)	Q	18-79
Lubben Social Network Scale (LSNS)	Q	18-79
EuroQol visual analogue scale (EQ VAS)	Q	18-79
Sense of Coherence Leipzig Short Scale (SOC-L9)	Q	18-79
Childhood Trauma Screener (CTS)	Q	18-79
Use of health care services	Q	18-79
Barratt’s Impulsivity Scale Version 11 (BIS-11)	Q	18-79
Behavioral inhibition system / behavioral activation system (BIS/BAS)	Q	18-79
NEO-Five-Factor Inventory (NEO-FFI-30)	Q	18-79
Need Inventory of Sensation Seeking (NISS)	Q	60-79
Hypomanic Personality Scale	Q	60-79
Sleep		
Pittsburgh Sleep Quality Index (PSQI)	Q	18-79
Multidimensional Fatigue Inventory (MFI-20)	Q	18-79
Epworth Sleepiness Scale (ESS)	Q	18-79
Morningness-Eveningness-Questionnaire (MEQ)	Q	18-79
Sleep Questionnaire (SF-A)	Q	60-79
Cognition		
Stroop test	T	18-79
Trail-Making Test A&B	T	18-79
Subjective Memory Impairment	I	18-79
Verbal Fluency Test ‘Animals’	T	18-79
Behavioral Assessment of the dysexecutive syndrome (DEX)	Q	18-79
Triangle test	T	60-79
Reading the mind in the eyes test	T	60-79
Structured Interview for Diagnosis of Dementia of Alzheimer type, Multi-infarct Dementia and Dementia of other Aetiology according to DSM-III-R, DSM-IV and ICD-10	I/T	60-79
Barthel-Index for basic Activities of Daily Living	I	60-79
Instrumental Activities of Daily Living Scales (IADL)	I	60-79
CERADplus neuropsychological test battery	T	60-79
Wechsler Memory Scale	T	60-79
Iowa gambling task	T	18-79^a^
n-back task	T	18-79^a^
Reversal learning task	T	18-79^a^
Depression		
Centre of Epidemiologic Studies - Depression Scale (CES-D)	Q	18-79
Childhood Trauma Questionnaire (CTQ)	Q	18-79
CIDI-DIA-X Screener	Q	18-79
Structured Clinical Interview for DSM Disorders (SCID)	I	60-79
Geriatric Depression Scale (15-item version, GDS-15)	Q	60-79
Inventory of Complicated Grief (ICG)	Q	60-79
Inventory of Depressive Symptoms (IDS-SR)	Q	60-79
Leipzig Life Event List	Q	60-79

^a^Only for the subgroup with additional obesity assessments

**Table 4:** Laboratory analyses

Parameter
Electrolytes: Sodium, Potassium, Chloride, Magnesium
Liver and pancreas: Alanine transaminase, Aspartate transaminase, Choline esterase, Gamma-glutamyltransferase, Bilirubin (total and direct), Lipase, Total protein, Albumin, Urea
Kidney: Creatinine, Cystatin C
Cardiac markers: Creatine kinase, Creatine kinase MB, Myoglobin, Troponine T high sensitive, N-terminal prohormone of brain natriuretic peptide
Lipid metabolism: Cholesterol, High density lipoprotein cholesterol, Low density lipoprotein cholesterol, Apolipoprotein B, Apolipoprotein A1, Trigycerides, Lipoprotein (a)
Glucose metabolism: Glucose, Insulin, C-peptide, Glycated hemoglobin (HbA1c)
Iron metabolism: Transferrin, Ferritin
Vitamins: Folic acid, Vitamin B12
Bone metabolism: Alkaline phosphatase, Phosphate, Calcium, Osteocalcine, Beta-CrossLaps, Propeptide of type I collagen, Parathormone, 25-Hydroxy vitamin D3
Endocrine function / hormones: Cortisol, Luteinizing hormone, Follicle stimulating hormone, Estradiol, Testosterone, Sex hormone-binding globulin, Dehydroepiandrosterone sulfate
Thyroid: Thyrotropin (TSH), Free triiodothyronine, Free thyroxine, TSH receptor antibodies, thyroglobulin antibodies, Thyreoperoxidase antibodies
Inflammatory mediators: Interleukin 6, C-reactive protein high sensitive
Allergy diagnostics: specific immunoglobulin E sx1 (timothy grass, rye, birch, mugwort, C. herbarum, D. pteronyssinus, cat, dog), fx5 (hen’s egg, cow’s milk, fish, wheat, peanut, soy), total immunoglobulin E
Hematology: Complete blood cell count with differential, Reticulocytes
Urine: Albumin, Creatinine, Immunoglobulin G

### Physical and medical examinations

#### Anthropometry

Only few anthropometric measures (e.g. body height, weight, waist, and hip) are usually obtained in epidemiological studies. It was our objective to investigate the potential of 3D body scanning technology to identify novel phenotypic anthropometric traits. Body scans of all participants were obtained using a laser-based 3D body scanner (Anthroscan VITUS SMART XXL with software ScanWorX 2.9.9b, Human Solutions) with 3 mm vertical and 1 mm horizontal resolution at 27 measurement points per square centimetre body surface. An initial set of about 150 length and circumference measures was computed from each scan.

Classical anthropometric measurements were also taken, according to standardized procedures by trained study nurses. Body weight was measured with an electronic scale (SECA 701, Seca Gmbh & Co KG) with a precision of 0.01 kg. Body height was assessed by means of a stadiometer (SECA 240) to the nearest 0.1 cm. Waist, hip, upper arm, thigh and calf circumferences were taken using an ergonomic circumference measuring tape (SECA 201) to the nearest 0.1 cm.

A subcohort of 1200 participants across all age groups was invited to undergo abdominal MRI in order to quantify visceral and subcutaneous adipose tissue volumes. The abdominal MRI scan covered the abdominal region starting 10 cm proximal and ending 10 cm distal from the umbilicus with a layer thickness of 1 cm. Semi-automated image segmentation was used to separate subcutaneous from visceral adipose tissues and to obtain volumetric data.

#### Heart function

It was our objective to obtain a deep phenotyping of the heart function to investigate various modes of heart failure and valve dysfunction and link this to coronary heart disease, carotid atherosclerosis and to vascular dementia in elderly participants. The aim was to identify and to follow subjects with first signs of reduced cardiac pump function. For this purpose cardiac ultrasound examination was performed using the GE Vivid 7 dimension BTO8 echocardiography station (GE Healthcare). Echocardiography was conducted by 3 study nurses, who were extensively trained for two months by a supervisor-sonographer with European certification. The assessment protocol lasted 20 min and included parasternal, apical, and subcostal views with 2D and apical view with 3D probes carefully described in an extensive SOP. In total, 17 sweeps were conducted in a highly standardized way and 28 images were taken (1 along parasternal axis, 7 in parasternal short axis, 8 in apical long axis including PW- and CW Doppler, TVI and TDI, 2 in apical 2 chamber view with TDI, 6 in apical 4 chamber view including TVI and TDI, 1 subcostal view, 3 other apical views). Standardized reading of the echocardiographic assessments was performed according to ASE recommendations and the European Society of Cardiology by means of the software EchoPAC Version 113 (GE Healthcare). Readings were performed by the sonographers and a trained reader. Structural parameters include ventricular, septal, and posterior wall thickness and left atrial and ventricular dimensions, which are used to calculate left ventricular volumes and mass, left ventricular ejection fraction, left atrial volume, and left atrial function. Readings were performed according to ASE recommendations and the European Society of Cardiology. Regarding diastolic dysfunction and heart failure, our method permitted to calculate the diastolic state by the assessment described by Nagueh et al. and Paulus et al [11, 12]. Analysis of the cardiac valves was performed according to the international guidelines by conventional 2D as well as color coded and Doppler echocardiography.

To investigate cardiac arrhythmias a 10-second 12-lead electrocardiogram (ECG) was recorded using the PageWriter TC50® ECG system (Philips Medical Systems) after a supine resting period of at least 10 min. The ECG was evaluated by means of the software ECGVue C.03.01.02 (Philips Medical Systems) based on published criteria with particular focus on rhythm and conduction disturbances, ST-segment and J-point changes, T and U waves, PQ and QT interval, hypertrophy, and QRS morphology [13–19].

#### Vascular system

It was a major objective to investigate and to follow early signs of atherosclerosis using extensive phenotyping of the vascular system. Blood pressure was measured three times at 3-min intervals using an automatic oscillometric blood pressure monitor (OMRON 705IT, OMRON Medizintechnik Handelsgesellschaft mbH) after resting for at least 5 min.

Using the same ultrasound device as for heart ultrasound, carotid artery sonography for atherosclerotic plaques and intima media thickness of the arterial wall were assessed in all participants. Atherosclerotic plaque of the carotid artery was defined according to the ‘American Society of Echocardiography Carotid Intima-Media Thickness Task Force’ as an echogenic thickening of intimal reflection that extends into the arterial lumen at least 0.5 mm or 50 % of the surrounding intima media thickness (IMT) of the common carotid artery (CCA) or an intimal plus medial thickness of >1.5 mm [20]. Assessment of central arterial stiffness and vascular aging was performed by non-invasive measurements of pulse-wave velocity (PWV), pulse wave analysis (PWA), augmentation index, and the ankle-brachial index (ABI) using oscillometry-, and photoplethysmography-based methods (Vicorder, Skidmore medical, UK).

#### Oral glucose tolerance test (OGTT)

It was our objective to determine the prevalence of unrecognized diabetes, to better understand mechanisms of insulin resistance, and to define a subcohort of prediabetic participants for future follow up. The OGTT test was conducted in all fasted participants who came to the study centre before 8 a.m. After initial blood drawing and an intake of 75 g of glucose solution (Accu-Chek® Dextrose O.G.T. Saft, Roche Diagnostics Deutschland GmbH), blood samples were taken for measurement of glucose and insulin concentration at 30 (glucose only) and 120 min.

#### Physical activity, fitness, and sleep patterns

Physical activity and sleep patterns are associated with health and hence were assessed quantitatively. Physical activity and sleep-wake regulation were objectively measured using a 2D-axial accelerometer. The SenseWear Pro 3 device (Bodymedia, Inc., Pittsburgh, PA) allowed recording of skin temperature, near body ambient temperature, heat flux, galvanic skin response, and motion. It is particularly useful for recording objective sleep duration. Participants were requested to wear the device continuously for 7 days and to keep a sleep and activity diary. Raw data were processed using the SenseWear Professional software to detect wake and sleep periods and to classify the time spent in low, moderate or vigorous physical activity, daily step counts and energy expenditure in MET-minutes. Maximal grip strength was assessed as the maximum of 6 alternate measurements of the right and the left hand using the Jamar dynamometer (Patterson Medical Ltd., Huthwaite, UK) [21].

#### Ophthalmological imaging

Degeneration of the macula and glaucoma are major age-related disorders associated with considerable handicap. It was our objective to unravel early signs of these disorders and follow them up longitudinally. *In-vivo* ophthalmological imaging techniques using OCT enabled sensitive detection of abnormalities. OCT is a medical diagnostic imaging technique which captures micron resolution 3-dimensional images. It is based on the principle of optical reflectometry. We employed OCT (Heidelberg Spectralis SD-OCT) and high quality non-mydriatic fundus imaging (Nidek AFC-230) to produce representative images of the retina and optic nerve. All images were read by two independent observers who classified degenerative changes and anomalies based on current ophthalmological standards.

#### Neuroimaging and neurocognitive assessments

Neurocognitive disorders are progressing particularly in the elderly. It was our objective to investigate early signs of neurodegeneration and of small vessel disease and their neurocognitive correlates. In addition we were interested whether obesity is mirrored in brain structures and functions. To obtain a structural and functional MRI assessment of the brain the following magnetic resonance pulse sequences were applied: i) Magnetization prepared rapid gradient echo using a 3D T1-weighted pulse sequence in order to assess brain structure and to highlight differences between grey and white matter. Based on these data, voxel-based morphometry and cortical thickness measurements were performed. ii) Diffusion weighted imaging at 60 different angles using pulse sequences, which are sensitive to water diffusion and its direction. Based on this information, parameters characterizing white matter such as various characteristics of the diffusion tensor (axial, radial diffusivity, fractional anisotropy etc.) were determined. iii) Resting state functional MRI. Here, oxygenation changes were investigated during rest as a measure for functional connectivity. iv) Fluid-attenuated inversion recovery, which is highly sensitive for the identification of white matter lesions. v) Susceptibility weighted imaging using a T2*-weighted pulse sequence, which is highly sensitive to brain hemorrhage. vi) MR-angiography, which was used for the assessment of arterial brain vessels, e.g. to identify aneurysms.

Structural and functional MRI parameters were used to identify specific neurodegenerative diseases and its pre-stages, subjective and mild cognitive impairment. For Alzheimer’s disease we focussed on atrophy in hippocampal regions and connectivity changes in temporoparietal brain networks, for subtypes of frontotemporal lobar degeneration on changes in structure and function of respective brain regions, for vascular diseases such as small vessel diseases on automatic detection of white matter lesions and changes in connectivity measures [22–25].

#### Electroencephalography

The onset of depression is believed to be linked to a dysregulation of the sleep-wake rhythm and a disturbance of vigilance regulation. According to the vigilance model of affective disorders put forward by Hegerl et al. [26] hyperstable vigilance regulation is associated with depressive disorders whereas unstable vigilance regulation is associated with disorders such as Attention Deficit/Hyperactivity Disorder or (hypo)manic states. To investigate this hypothesis for the first time in a large population-based cohort elderly participants underwent an EEG comprising of 3 paradigms: A 20-min resting EEG was performed to quantify wakefulness (EEG-vigilance) regulation according to the Leipzig Vigilance Algorithm Protocol (VIGALL, http*:*//uni-leipzig.de/*~*vigall/). Furthermore, evoked potentials were assessed using a 15 min Novelty-Oddball-Paradigm (P300), and a 15 min Intensity Dependence of Auditory Evoked Potentials paradigm [27, 28]. Alterations in P300-amplitude and latency have been linked to neurodegenerative processes and IAEP-magnitude has been linked to serotonin levels in the brain [28, 29]. All EEG recordings were performed by trained staff in an electrically shielded and sound attenuated EEG booth using a 31-channel EEG (QuickAmp, Brain Products GmbH). Instructions were standardized using presentation scripts (Presentation Software).

#### Voice function

The voice is a highly individual part of the human personality and a base of speech development and communication. It was our objective to phenotype the human voice by standardized voice limit measurements (frequency dependent sound-pressure levels) when speaking and singing. This provides a profile of the volume and pitch range of a voice [30]. Profiling was undertaken as a novel phenotype assessment in an epidemiological setting. So far only twin studies are available [31]. The speaking voice is examined from quietest speaking over two raised loudness levels up to shouting while continuously counting. A microphone was attached to a headset at a distance of 30 cm from the mouth (DiVAS® device, XION medical, Berlin, Germany). The measurement of the singing voice included softest and loudest singing, each beginning in mean fundamental frequency of speaking, then singing stepwise down to the lowest and up to the highest frequency. The measurement values and their registration are displayed as a speaking and a singing voice profile. An acoustic analysis (real-time spectrum) for each individual tone provided additional information about the resonance quality of the voice.

#### Olfactory test

Epidemiological data on the olfactory function in larger and representative samples of the general population are scarce. It was our objective to investigate whether olfactory function is an early indicator for neurodegeneration and possibly cognitive impairment. In order to assess the ability to identify different odours, the Burghart Sniffin’ Sticks “Screening12 Test” (Burghart Messtechnik GmbH, Wedel, Germany) was applied using 12 felt-tip pens. The odours of these sticks were peppermint, fish, coffee, banana, orange, rose, lemon, pineapple, cinnamon, cloves, leather and liquorice. Participants were asked to name the odour as a forced choice out of four possible answers, only one of which was correct.

#### Allergy test

It was our objective to investigate allergy prevalence in adult patients of advanced age and to investigate associations with immunological signals in the blood. Subjects of all age groups underwent skin prick test (ALK-Abello, Hamburg, Germany) with positive/negative control, birch, timothy grass, mugwort, ragweed, alternaria, and *Dermatophagoides pteronyssinus*. Reading was performed after 15 min either by ruler measurement or by a three dimensional imaging system (Primos-Pico, Fa. GFMesstechnik, Teltow; software from red.soft it-service GmbH Oberpullendorf, Austria) [32, 33]. Furthermore, measurement of IgE was performed (total/sx1: timothy grass, rye, mugwort, birch, Cladosporium herbarum, Dermatophagoides pternonyssinus, cat, dog/fx5: hen’s egg, cow’s milk, codfish, wheat flour, peanut, soy).

### Interviews, questionnaires, and cognitive tests

#### Socio-economic status

Data on socio-economic and socio-demographic factors were obtained in a structured interview. A multidimensional index was calculated from information on education (school, professional), occupational status, and equivalent household income [34, 35].

#### Medical history, family history, and medication

Participants were interviewed about medical diagnoses that were previously confirmed by a physician, including age at first diagnosis, whether symptoms were present in the past 12 months, and whether the disease was currently under treatment. The interview comprised over 70 common diseases. In addition, participants were interviewed about diseases in their first degree relatives. Data on medications and dietary supplements taken within the last 7 days before the core programme visit were gathered. Medication was identified by bar codes scanned from the original packaging presented by the participants. For data analysis, all medications were coded by the active agents using the Anatomical Therapeutic Chemical classification system.

#### Lifestyle and diet

The tobacco assessment contained questions on ever and current smoking, duration of smoking, amounts of different tobacco products, and exposure to passive smoking. Alcohol consumption was assessed with regard to frequency and amount of different alcoholic beverages consumed within the last year, as well as on alcohol abuse in the past. Regarding dietary patterns, participants were asked about the consumption frequency of 34 food groups and 13 types of beverages within the last 12 months. Questions related to the vegetarian diet, meals and body weight were asked. Specific aspects of eating behaviour such as cognitive restraint, disinhibition, and hunger as well as the frequency of common difficulties in eating behaviour in the general population (e.g. craving for sweets, eating in response to stress) were assessed using the German version of the Three-Factor-Eating Questionnaire [36]. Subjectively reported physical activity was assessed using the short form of the International Physical Activity Questionnaire (IPAQ-SF, https://sites.google.com/site/theipaq/).

#### Cognitive performance

To assess the prevalence of cognitive impairment, to investigate putative risk and protective factors of dementia and corresponding pre-stages such as subjective memory impairment and mild cognitive impairment, and to analyze general cognitive functioning in the population. Several subjective and objective cognitive performance assessments were examined in all participants. Subjective cognitive performance was assessed via a structured 15-item interview. Objective cognitive performance was assessed using – amongst others – the Trail-Making Test A & B and the Verbal Fluency Test ‘Animals’, which are subtests of the CERADplus test battery (extended neuropsychological test battery of the Consortium to Establish a Registry for Alzheimer’s Disease) [37, 38]. Elderly participants underwent an additional deep and comprehensive cognitive assessment programme on a separate day (cognition programme) together with a brain MRI-scan. The programme included, for example, all the further subtests of the CERADplus test battery as well as the SIDAM (Structured Interview for Diagnosis of Dementia of Alzheimer type, Multi-infarct Dementia and Dementia of other Aetiology according to DSM-III-R, DSM-IV and ICD-10) [39]. Impairments in daily activities were assessed via the Barthel-Index (basic activities) and the Instrumental Activities of Daily Living Scale (IADL) [40, 41].

#### Depressive symptoms

Depressive symptoms are the most common mental symptoms in the population. The seriousness of depressive symptoms becomes apparent in the burden that comes along with such symptoms including incapacity to work and high health care costs. Our objective was to determine the prevalence of depressive symptoms in the general population and to identify subgroups that are especially vulnerable to suffer from depressive symptoms. Depressive symptoms were assessed in all probands using the German version of the Centre of Epidemiological Depression Scale (CES-D) screening instrument [42, 43]. In the elderly subgroup, a more detailed assessment of depressive symptoms was performed (depression programme). Additional self-ratings were taken using the Inventory of Depressive Symptoms (IDS-SR) and the Geriatric Depression Scale (GDS-15) [44, 45]. In all elderly persons the diagnosis of psychiatric illnesses was assessed using the Structured clinical interview for DSM-IV (SCID-I) [46].

#### Psychosocial aspects, life satisfaction, stressors

It was our objective to determine the impact of psychosocial factors and personality profiles on multiple health conditions. In addition we aimed to derive new normative values of the general population for novel instruments, based on a large community-based sample, and to investigate the relationship between quality of life, psychosocial factors, socio-demographic variables, and health behaviour. We therefore applied a broad battery of self-reported assessments including instruments measuring quality of life, personality, and several psychosocial factors. In particular, the following questionnaires were used: The Short Form 8 (SF-8) measuring health-related quality of life [47], the Generalized Anxiety Disorder questionnaire (GAD-7) [48], the Patient Health Questionnaire-15 (PHQ-15), measuring bodily complaints [49], the ENRICHD Social Support Scale (ESSI) [50], the Life Orientation Test – revised (LOT-R), measuring dispositional optimism [51], the Satisfaction With Life Scale (SWLS) [52], the Personality Adjective List (16-AM), measuring personality traits according to the Big Five [53], the Lubben Social Network Scale (LSNS) [54], the EuroQol visual analogue scale (EQ VAS) [55], the Sense of Coherence Leipzig Short Scale (SOC-L9) measuring a global orientation to view the world and the individual environment [56], and a short form of the Childhood Trauma Questionnaire (CTQ) – the Childhood Trauma Screener (CTS) – as a scale for the retrospective assessment of childhood abuse and neglect experiences [57, 58].

#### Sleep and sleepiness

It was our objective to investigate the role of sleep duration (see above) and sleep quality for somatic and mental health. A short sleep duration is associated with weight increase, obesity, diabetes type 2, hypertension, and overall increased morbidity and mortality [59–66]. Sleep problems are also closely associated with psychiatric conditions, especially affective disorders [67–71]. All participants were asked to evaluate their sleep quality and other sleep-related aspects using standardized questionnaires: e.g. the Pittsburgh Sleep Quality Index (PSQI) [72], and the Epworth Sleepiness Scale (ESS) [73] (for details see Table 4).

### Laboratory measurements

It was our objective to unravel and to validate laboratory biomarkers significantly associated with disease phenotypes and risks. An extensive panel of laboratory tests covering 83 analytes and biomarkers (clinical chemistry, hematology, immunology, endocrinology and metabolism) was performed on fresh biospecimen directly on the day of sample collection in a highly standardized manner (Table 4). It is a major goal to investigate and to identify novel genetic modifiers of phenotypes and disease risk. To this end, we aimed to genotype all participants using the Affymetrix AXIOM-CEU genome-wide SNP array, addressing a total of 587,352 variants. Genotyping was accompanied by genome-wide gene expression analyses for the whole blood, which was collected in Tempus Blood RNA Tubes (Life Technologies) and transferred to -80 °C prior to further processing. Isolated RNA was processed and hybridised to Illumina HT-12 v4 Expression BeadChips (Illumina, San Diego, CA, USA) and scanned on the Illumina HiScan. We investigated the association of metabolic biomarkers (quantitiative tandem mass spectrometry: amino acidy, fatty acid oxidation, steroids, sterols, eicosanoids, phospholipids, tri- and diacylglycerols, apolipoproteins and others) with major disease phenotypes, the genome and other biomarkers. For the whole blood analysis of 26 amino acids, free carnitine and 34 acylcarnitines, 40 μl of native EDTA whole blood were spotted on filter paper WS 903 (GE Healthcare, Germany). Dried blood spots were stored at -80 °C after 3 h of drying until batch analysis. Sample pretreatment and measurement are described in detail elsewhere [74, 75]. Analyses were performed on an API 2000 and API 4000 tandem mass spectrometer (Applied Biosystems, Germany). Quantification was performed using ChemoView™ 1.4.2 software (Applied Biosystems, Germany).

In a subcohort of over 900 participants comprising all age groups, a detailed leukocyte subtype phenotyping with an extensive antibody panel was performed from EDTA whole blood samples using 10 colour flow cytometry (Navios flow cytometer, Beckman Coulter, Pasadena, CA, USA) [76, 77].

## Discussion

Before the main study recruitment started we had undertaken a series of feasibility studies to investigate the suitability of assessment procedures and to define SOPs. We then defined the parcours of all assessments and conducted a full pilot study with 400 volunteer participants. This helped us to optimize the logistics and the timing of the main study.

Recruitment for the main study started in August 2011 and ended in November 2014 when the planned sample size of 10,000 participants was reached. During the first 13 months of the recruitment phase, the study centre was located in a provisional building with limited space. Therefore, recruitment was restricted to 8-10 participants per day. In September 2012 the study site moved to a more spacious and reconstructed building and recruitment could be increased to 18-20 participants per day (7 am to 4.30 pm, Monday to Friday). Once per month the study centre was in operation on Saturday in order to allow participation of individuals who were not able to attend on a weekday. Assessments were performed by trained study nurses and interviewers. One staff member was responsible for quality management. Participants were informed about the the assessment programme by watching a video, which was shown in the waiting area.

The study programme attracted high public attention. Recruitment was accompanied by reports in local newspapers and on local television to increase awareness. A website was established with information and video trailers (http://life.uni-leipzig.de). There were annual press releases with status reports. several open days were organised, which were well attended. Among all invited citizens from the city of Leipzig aged 40 or older, 33 % did actively participate, 30 % declined to participate, and 37 % did not respond despite a reminder letter. Efforts were undertaken to obtain telephone numbers and to contact non-responders by telephone. However, telephone numbers for only 25 % of non-responders could be retrieved from publicly available telephone directories. Of these, only 18 % finally agreed to participate, contributing 4 % of the response rate.

The core assessment programme was planned to last 5-6 h. Except for some of the above mentioned elderly participants over the age of 75, almost all participants successfully completed the entire assessment programme as planned. A few physical and medical assessments were performed only in subsamples due to logistic reasons (e.g., oGTT tests were only conducted in participants scheduled in the morning before 8 a.m.) or because they were added to the assessment programme at a later phase during the study (see Table 3 for details). An evaluation of a questionnaire on participants’ satisfaction with the study showed that 96.0 % of the participants were very satisfied or satisfied. A total of 88.5 % stated that they would do the assessment again and that they would recommend it to other participants. Only 1.8 % of the participants stated that they would not participate again. Compliance with the extended cognition and depression programmes for the elderly participants was also high.

No participant showed a severe pathological finding that would have required immediate intervention. Fifteen participants had findings that were not acute life-threatening but required immediate medical clarification or treatment. Furthermore, 116 participants were informed by the study centre to seek for near-term medical clarification based on pathological measurement results.

For MRI assessments, very strict inclusion and exclusion criteria applied. One exclusion criterion was the presence of skin tattoos due to the potential risk of cutaneous burns due to ferromagnetic compounds in tattoo pigments. We observed a high prevalence of skin tattoos in persons below 40 years (approximately 50 %). This decreased the participation rate in the younger obesity sub-cohort remarkably.

The LIFE-Adult-Study is so far the largest population-based study of urban individuals with such extensive phenotyping conducted in Germany. It is building on the experience of other population studies which have been conducted in Germany in the past, such as the KORA, SHIP, CARLA and Heinz-Nixdorf-Recall studies [2–6]. In the design and implementation phase of the LIFE-Adult-Study extensive interactions and support from these German study teams helped to define the comparability of the assessments and SOPs. Principle investigators of these studies are members of the scientific advisory board of LIFE.

A particular strength of the LIFE-Adult-Study is the deep and innovative phenotyping in several fields of interest strongly supported by researchers from the Medical Faculty of the University of Leipzig. A particular objective in the field of neurodegenerative diseases is the multidimensional assessment of mild cognitive impairment, which often is a pre-stage of clinical manifest dementia. This is investigated by multiple cognitive tests complemented by a high resolution 3 T-MRI scan regarding brain morphology, resting state and DTI. Another objective of particular concern is the deep assessment of depressive symptoms and whether they relate to clinically diagnosed manifest depression as measured by the SCID instrument. To investigate the link to sleep-wake regulation this is accompanied by investigations of sleep quality and rhythm and an EEG-based paradigm assessment of vigilance regulation. Metabolic disorders relating to glucose and lipid metabolism, and associated diseases like obesity and the metabolic syndromes are another major interest in the LIFE-Adult-Study. This led us to undertake extensive laboratory investigations including metabolic and extensive heart and vascular examinations notably with regard to atherosclerotic phenotypes. In addition, participants were asked for social isolation, life style and quality of life. For the first time in an epidemiological setting, we used 3D body scans to assess body shapes which permits to define novel anthropometric phenotypes in much greater detail than classical anthropometry is able to provide. Particular attention is paid to early degenerative changes of the retina and the optic nerve region. We performed one of the first population based assessment employing OCT to unravel early-onset lesions of the retina.

At present we have started the data curation process of the entire cohort database. We are performing and completing the reading of the signal and image based data (e.g. MRI-scans, EEG-scans, ECG-recordings, OCT-images, heart ultrasound, carotis examination, voice profiles and accelerometer read-outs). A first wave of SNP-based genotyping of 4500 participants will be available soon. We expect to have prevalence estimates of major clinical and subclinical phenotypes by autumn 2015.

The LIFE-Adult-Study is designed as a longitudinal cohort study. Based on a first interim analysis of 4300 participants in 2013 we can project a conservative calculation on expected incident cases. At a follow-up after 4 years we expect numerous newly diagnosed events such as diabetes (~200 cases), hypertension requiring treatment (~500 cases), severe heart failure (~300), participants with vascular alterations in the carotids (~500), pathological macula lesion (~150), cutaneous allergies (~300), and dementia (~80). Based on the data from all 10,000 participants we will design details of the first longitudial follow-up scheduled to start in 2016.

The LIFE Centre for Civilization Diseases is also conducting other cohort and patient studies. The LIFE-Child-Study is currently recruiting a neonate sub-cohort and a population based cohort of children and adolescents in an age range from 3-18 years. This study is recalling participants in a narrow time frame of 1-2 years [78]. The LIFE-Heart-Study is a clinical cohort of heart patients with clinical indication for coronary angiography. It consists of a large subcohort of patients with first initial angiography of the coronary arteries and a subcohort of acute myocardial infarction survivors [79]. All patients of the LIFE-Heart-Study are recruited (7000 participants). The study belongs to the largest fully genotyped studies worldwide with angiographically assessed coronary patients. The LIFE-Heart-Study is a collaborating member of large international networks for genome metanalysis (Cardiogram, Genius). Since 2014 the LIFE study centre is presently recruiting new participants as a regional study site of the German National Cohort, which has recently started and aims to set a nationwide cohort of 200,000 individuals [80].

Here we have reported about the design of the study to provide the community with this information, to facilitate subsequent forthcoming publications and reach potential future cooperation partners.

## Abbreviatons

ABI: Ankle brachial index
CCA: Common carotid artery
DNA: Deoxyribonucleic acid
ECG: Electrocardiogram
EEG: Electroencephalography
IMT: Intima media thickness
LIFE: Leipzig Research Centre for Civilization Diseases
LIMS: Laboratory information management system
LIO: LIFE information ontology
MRI: Magnetic resonance imaging
OCT: Optical coherence tomography
OGTT: Oral glucose tolerance test
PBMC: Peripheral blood mononuclear cell
PWV: Pulse wave velocity
RNA: Ribonucleic acid
SOP: Standard operating procedure
